# [Retracted] SMAD specific E3 ubiquitin protein ligase 1 promotes ovarian cancer cell migration and invasion via the activation of the RhoA/ROCK signaling pathway

**DOI:** 10.3892/or.2025.8938

**Published:** 2025-06-30

**Authors:** Wei Wang, Han Du, Huiping Liu, Fangfang Hu, Guangzhi Liu

Oncol Rep 41: 668–676, 2019; DOI: 10.3892/or.2018.6836

Following the publication of this paper, it was drawn to the Editor's attention by a concerned reader that a pair of the data panels shown for the Transwell migration assay experiments in Fig. 1B on p. 671, and two further pairs of data panels showing the results of invasion and migration assay experiments in Fig. 2A and B on p. 672 respectively, contained overlapping sections of data, such that the affected panels, which were intended to show the results of differently performed experiments, had all apparently been derived from the same original sources.

Owing to the identification of these duplicated data panels in this paper, the Editor of *Oncology Reports* has decided that it should be retracted from the Journal on account of a lack of confidence in the presented data. The authors were asked for an explanation to account for these concerns, but the Editorial Office did not receive a reply. The Editor apologizes to the readership for any inconvenience caused.

